# Cuticular wax profiling of *Populus trichocarpa* and *P. balsamifera* reveals surface similarities with underlying differences

**DOI:** 10.3389/fpls.2026.1846385

**Published:** 2026-06-11

**Authors:** Mahbobeh Zamani-Babgohari, Huiru Peng, Armando Geraldes, Charles A. Hefer, Jessica Y. Hu, Raju Y. Soolanayakanahally, Shawn D. Mansfield, Eliana Gonzales-Vigil

**Affiliations:** 1 Department of Cell and Systems Biology, University of Toronto, Toronto, ON, Canada; 2 Department of Biological Sciences, University of Toronto Scarborough, Scarborough, ON, Canada; 3 Department of Wood Science, Faculty of Forestry, University of British Columbia, Vancouver, BC, Canada; 4 Department of Zoology, University of British Columbia, Vancouver, BC, Canada; 5 Bioinformatics, Analytics and Modeling, Bioeconomy Science Institute, Agresearch Group, Lincoln, New Zealand; 6 Indian Head Research Farm, Agriculture and Agri-Food Canada, Indian Head, SK, Canada; 7 Department of Botany, Faculty of Science, University of British Columbia, Vancouver, BC, Canada

**Keywords:** alkenes, cuticular waxes, GWAS, phenolics, poplar, *Populus balsamifera*, *Populus trichocarpa*

## Abstract

Cuticular waxes are a mixture of hydrophobic components protecting plant tissues from the environment. *Populus trichocarpa* and *P. balsamifera* are closely-related tree species with broad North American distribution; however, variation in their cuticular wax composition across their range remained poorly understood. To address this gap, stem and leaf waxes from both species across three developmental stages were profiled by gas chromatography-mass spectrometry. A core set of compounds shared across tissues, accessions, and developmental stages was detected, as well as tissue- or species-specific constituents such as phenolics and alkenes. Phenolic-derived compounds were more prevalent at early stages of development compared to the predominantly aliphatic composition of mature tissues. Moreover, early stage leaves also showed enhanced protection from desiccation and oxidative stress in a subset of accessions. Alkenes, found exclusively in leaves, segregated the poplar accessions into alkene-producing and alkene-lacking phenotypes. GWAS analysis of 174 P*. trichocarpa* accessions identified a region on chromosome 10 containing a tandem cluster of ketoacyl CoA synthases associated with alkene accumulation in *P. trichocarpa*, which was not significantly associated in 133* P. balsamifera* individuals examined. These findings reveal dynamic, developmentally regulated, and species-specific variation in poplar wax profiles. Understanding these chemical traits offers new opportunities to develop *Populus* varieties with enhanced environmental resilience and adaptive potential.

## 1 Introduction

The genus *Populus* includes economically and ecologically important tree species distributed across the Northern Hemisphere, with wide latitudinal, altitudinal and longitudinal variation (Cronk, 2005). *Populus trichocarpa* Torr. & Gray (black cottonwood) is found latitudinally along the coast west of the Rocky Mountains, from California to southern Alaska, and is adapted to the mild and humid conditions found in riparian habitats. *P. balsamifera* L. (balsam poplar) is parapatric to *P. trichocarpa*, distributed longitudinally from Northern Alaska to the east Coast of Canada. It has adapted to the harsher environmental conditions of boreal forests, including lower annual precipitation (Suarez-Gonzalez et al., 2018). Despite their distinct niche preferences, their genomes reveal a recent divergence from a common ancestor (~76 Ka) with approximately 56% of Single Nucleotide Polymorphisms (SNPs) shared between them (Geraldes et al., 2014; Levsen et al., 2012).

Poplar population structure is shaped by demographic history and local adaptation. Using ~400 SNPs, 474 individuals of *P. balsamifera* were grouped into three distinct demes: Northern, Central and Eastern (Keller et al., 2010). The Central deme, characterized by a higher level of heterozygosity and larger effective population size, is thought to be the closest to the ancestral population. The Eastern and Northern demes likely emerged from a recent expansion after glacial retreat (18 Ka), and continue to receive substantial gene flow from the Central populations (Keller et al., 2010). Similarly, a 34K SNP phenotyping array identified four geographic clusters with independent ancestry in *P. trichocarpa*: Northern British Columbia (BC), Central interior BC, Southern BC, and a separate cluster from Oregon in the United States (Geraldes et al., 2014). Interestingly, hybridization between the species can increase genetic diversity, particularly in river drainages at the edges of *P. trichocarpa* distribution in Northern and Central interior BC (Can, 2022; Geraldes et al., 2014; Suarez-Gonzalez et al., 2018). This is illustrated in a 880-kb region on chromosome 15 that introgressed from *P. balsamifera* into *P. trichocarpa* that shows strong signatures of selection (Geraldes et al., 2014; Suarez-Gonzalez et al., 2018). The broad geographic range and high genetic diversity make *P. trichocarpa* and *P. balsamifera* excellent models for studying how environmental factors influence protective chemical traits like the cuticle.

The cuticle consists of a cutin matrix interspersed with cuticular waxes deposited on top and within covering all aerial surfaces of plants (Kunst and Samuels, 2009). Cuticular waxes are predominantly composed of aliphatic derivatives of very-long-chain fatty acids (VLCFAs, more than 18 carbons in length). Aliphatics can be accompanied by phenolic-derived compounds, triterpenoids, and compounds combining a phenolic core with an aliphatic tail, such as alkyl hydroxycinnamates (AHCs) (Jetter et al., 2006; Chen et al., 2011; Domergue and Kosma, 2017; Nomberg et al., 2022). The biosynthesis of aliphatic waxes starts with the action of the Fatty Acid Elongation complex consisting of four enzymes that take a C16 or C18 CoA-bound fatty acid and malonyl CoA to produce an acyl-CoA two carbons longer than the original substrate (Haslam and Kunst, 2013; Li-Beisson et al., 2013). The beta ketoacyl CoA synthase (KCS) is the first enzyme in the complex and determines what acyl CoA primer, in terms of length, unsaturation and stereochemistry, is used for condensation with malonyl CoA (Chen et al., 2022). Iterative cycles of elongation are required to generate fatty acids up to 34-carbons in length that are functionalized through three different pathways in *Populus*: the alcohol-forming pathway that produces primary alcohols and wax esters, the alkane-forming pathway that produces alkanes, aldehydes, ketones and secondary alcohols, and the enoic pathway that is responsible for the production of alkenes (Chen et al., 2024; Delude et al., 2016; von Wettstein-Knowles, 2007).

Although wax biosynthesis has been extensively characterized in other species, only a few genes have been implicated in *Populus* (Cheng et al., 2013; Gonzales-Vigil et al., 2021, 2017; Meng et al., 2019; Molina et al., 2009; Molina-Rueda et al., 2013; Xue et al., 2015). Among these, the expression of a KCS (*PtKCS1)* was found to be highly predictive of the accumulation of *cis* 9-alkenes (herein referred to as alkenes) in *P. trichocarpa*. Natural accessions of *P. trichocarpa* segregate into two phenotypic groups depending on alkene accumulation on leaves: Alkene Plus (AP) accessions exhibit high *PtKCS1* expression and accumulate alkenes, and Alkene Minus (AM) accessions show low expression and lack alkenes (Gonzales-Vigil et al., 2017). PtKCS1 has preference for using *cis* 9 monounsaturated fatty acids for elongation, which can then be decarbonylated or decarboxylated to alkenes that accumulate in fully expanded leaves of *P. trichocarpa* AP accessions.

The cuticle chemistry supports its various functions, primarily reducing non-stomatal water loss from leaves (Fernández et al., 2016). Physicochemical studies suggest that water loss is conferred by the lipophilic composition of the cuticle, whereases polysaccharides embedded in the cutin matrix are potentially responsible for water retention (Chamel et al., 1991; Tredenick and Farquhar, 2021). In addition to this role, the cuticle protects plant cells from external abiotic and biotic stressors, such as radiation, pathogens and insect herbivores. In response to UV-B radiation, the cuticle deposits antioxidant phenolic compounds like *p*-coumaric acids and chalcones shielding internal tissues from damaging photons and oxidative stress (Bringe et al., 2007; González Moreno et al., 2022; Kakani et al., 2003; Steinmüller and Tevini, 1985). To fulfill its diverse roles, the cuticle can adjust its thickness, composition, and load across tissues, growth stages and environmental conditions (Tredenick and Farquhar, 2021). Correspondingly, the cuticles of *Populus* are highly variable across species, and within tissues of the same tree (Chen et al., 2023; Gonzales-Vigil et al., 2017; Rains et al., 2018; Xu et al., 2016). There are also compositional differences between leaf surfaces, as AP accessions of *P. trichocarpa* have been shown to accumulate alkenes only on the abaxial side (Gonzales-Vigil et al., 2017).

In this study, we analyzed stem and leaf wax composition across developmental stages in two *Populus* species to explore the factors driving variation in cuticular waxes. We found substantial variation within and between *P. balsamifera* and *P. trichocarpa*, with young leaves showing enhanced protection against water loss and oxidative stress. Next, we conducted GWAS to uncover the genetic basis of alkene accumulation. We identified SNPs around *PtKCS1* that predict alkene accumulation in *P. trichocarpa*, but not in *P. balsamifera*. Collectively, these results enhance our understanding of the variation in cuticular waxes in long-lived and widely distributed forest species. The knowledge can lead to more efficient selection of adaptive traits for future climate scenarios.

## 2 Materials and methods

### 2.1 Cuticular wax analysis

After fulfilling their chilling requirements, dormant cuttings of 16 accessions of *P. balsamifera* (RRID: NCBITaxon_3694) representing AM and AP chemotypes (six and ten individuals, respectively) were collected from the Agriculture Canada Balsam Poplar (AgCanBaP) collection at the Indian Head Research Farm, Agriculture and Agri-Food Canada in Saskatchewan (Soolanayakanahally et al., 2013). In contrast, four accessions of *P. trichocarpa* (2 AM and 2 AP) were initially sourced from the Totem Field common garden (University of British Columbia, Vancouver, 49°15’22.6”N 123°14’58.3”W), propagated via tissue culture, and subsequently transferred to soil after successful root development. Representatives of both species were grown in a growth chamber at University of Toronto Scarborough, with a 16-h photoperiod, at 23°C and 40% RH for three months. Young leaves of plastochron index (PI) number four (± 1) were sampled, from each, five leaf discs of 10 mm in diameter were collected. Stem samples were taken from three developmental stages where the internodes between leaves six, seven and eight corresponded to the young stage, between leaves 10, 11, and 12 to the intermediate stage and 14, 15, and 16 to the mature stage. Leaves were removed before using the peeled bark for chemical analyses. Following wax extraction, the surface area of the stem pieces was measured with Image J (Schneider et al., 2012) from pictures taken with a scale bar. Two or three trees were sampled from each accession.

To determine the wax composition, cuticular waxes were extracted by dipping leaf and stem pieces in chloroform containing 10 µg of the internal standard n-tetracosane for 30 seconds, as described previously (Gonzales-Vigil et al., 2017). Derivatized waxes were analyzed on an Agilent 5977A Series GC-MSD fitted with a 30 m × 0.25 mm × 0.25 μm HP-5 column. Waxes were separated with the following temperature program: 50°C for 2 min, increased to 200°C by 40°C/min, maintained at 200°C for 1 min, increased at 320°C by 3°C/min, and then maintained at 320°C for 10 min. Wax components were identified by comparing their mass spectra with those available in the NIST-17 (National Institute of Standards and Technology) library. Peak areas for all wax components were quantified using the MassHunter Quantitative Analysis software with specific quantifier ions for each compound class (Agilent, V5). Data were normalized based on the internal standard, and by surface area (cm^2^).

### 2.2 Leaf water loss assay

To measure water loss in excised leaves, four individuals were chosen: WHR3 (AM-Alkene minus) and MTN9 (AP-Alkene plus) from *P. balsamifera*, and BELA18-4 (AM) and BELC18-1 (AP) from *P. trichocarpa*. Leaves from two developmental stages (young and mature) were collected and their fresh weight was immediately recorded. The excised petioles were sealed with melted parafilm before placing the leaves in the dark at 20-23°C and 45% RH for 6 h. Leaves were weighed every two hours for six hours and then placed at 60°C until the dry weight was constant. Water loss was calculated based on the following formula:


$$Water\;loss\;\left(\%\right)=\left[\frac{FW-Wt}{FW-DW}\right]\times 100$$

where, *FW* is the fresh weight, *Wt* represents the weight of the leaf after a defined period of dehydration, and *DW* stands for the final dry weight (Hasanuzzaman et al., 2017).

### 2.3 Antioxidant activity of cuticular wax extracts

The antioxidant activity of the wax extract was measured using 2,2-diphenyl-1-picrylhydrazyl (DPPH) by adapting the method of Yen and Duh (1994). First, cuticular waxes from the same four accessions used for the water loss assay were extracted by dipping three leaf discs in chloroform for 30 sec. The resultant extracts were evaporated to dryness and dissolved in 4 mL of methanol for subsequent antioxidant assay. A calibration curve was established using butylated hydroxytoluene (BHT) as standard. Briefly, 20 µl of BHT standard at 10, 20, 40, 80 and 100 mg/L was mixed with 180 µl of 0.05 mM DPPH and kept at room temperature in the dark for 30 min before measuring the absorbance at 517 nm. To measure the antioxidant activity of the leaf samples, 10 µl of the wax extract was mixed with 190 µl of the 0.05 mM DPPH and placed in the dark before measuring. Absorbance was monitored at time intervals up to 72 hours.

The DPPH scavenging activity was calculated based on the following equation:


$$DPPH\;scavenging\;activity\;\left(\%\right)=\frac{A_{0}-A_{1}}{A_{0}}\times 100$$

where, *A_0_* is the absorbance of the blank, and *A_1_* is the absorbance of the extracts/standard.

### 2.4 Screen of cuticular waxes from *P. balsamifera* collection

For the larger screen of *P. balsamifera* accessions, leaves were collected from a common garden housed at University of British Columbia Totem Field, which is part of AgCanBaP collection (Soolanayakanahally et al., 2013). During Spring 2015, 160 accessions of *P. balsamifera* were sampled. From each accession, two leaves at PI seven were taken from different branches (two replicates per tree). From each leaf, two leaf discs 13 mm in diameter were placed in microcentrifuge tubes, immediately stored in liquid nitrogen, and subsequently transferred to -80°C. Cuticular waxes were extracted as described above and run on a GC-FID equipped with a 30 m × 0.32 mm × 0.1 μm film HP-1 column. Peak areas were normalized by the internal standard and leaf area for further analysis as previously mentioned. Peaks that were absent from more than 10% of samples were removed from analysis.

### 2.5 Genome-wide association analysis

The differences in *PtKCS1* expression and alkene accumulation between *P. trichocarpa* accessions suggested that alkene accumulation behaved as a binary trait that was either present or absent (Gonzales-Vigil et al., 2017). Hence here, GWAS was performed to identify SNPs that differentiate alkene-containing (AP) and alkene-lacking (AM) accessions. Genomic data from SRA projects PRJNA276056 and PRJNA298917, following the SNP identification method described in (Geraldes et al., 2015) for 174 accessions of *P. trichocarpa* (144 AP and 30 AM) was filtered to include only SNPs (insertion and deletion variants eliminated) that are biallelic (two alleles only), no more than 10% missing genotypes (minimum genotype quality of 10, *i.e.* an error rate of less than 10%) per locus and with a minor allele frequency of 1% or higher resulting in 9,124,598 SNPs. Additionally, we eliminated 0.9% of SNPs with an observed heterozygosity higher than 0.6, as they have a high likelihood of not being allelic variants at a single locus but instead reflecting fixed differences between paralogs. Association analysis was performed in Plink with 9,038,432 SNPs after a permutation test revealed that AM and AP accessions are not less similar than expected (*p*-value 0.627). The same analysis was performed independently for *P. balsamifera* (60 AP and 78 AM) with SNPs filtered in the same manner as above for *P. trichocarpa.*

### 2.6 Heterologous expression of *PtKCS1* in yeast

A synthetic gene based on the previously characterized Potri.010G079500 from HALS30-6 (AP) modified at five positions to incorporate the non-synonymous SNPs identified in AM accessions was ordered from Twist Bioscience. The modified AM-PtKCS1 was cloned into pYES-DEST52 and expressed as described elsewhere (Gonzales-Vigil et al., 2017). The VLCFAs profile of the original “AP-PtKCS1”, modified “AM-PtKCS1” and the empty control vector were compared by GC-MS analysis (Chen et al., 2022).

## 3 Results

### 3.1 Large chemical variation in wax accumulation in poplars

**Figure 1:**
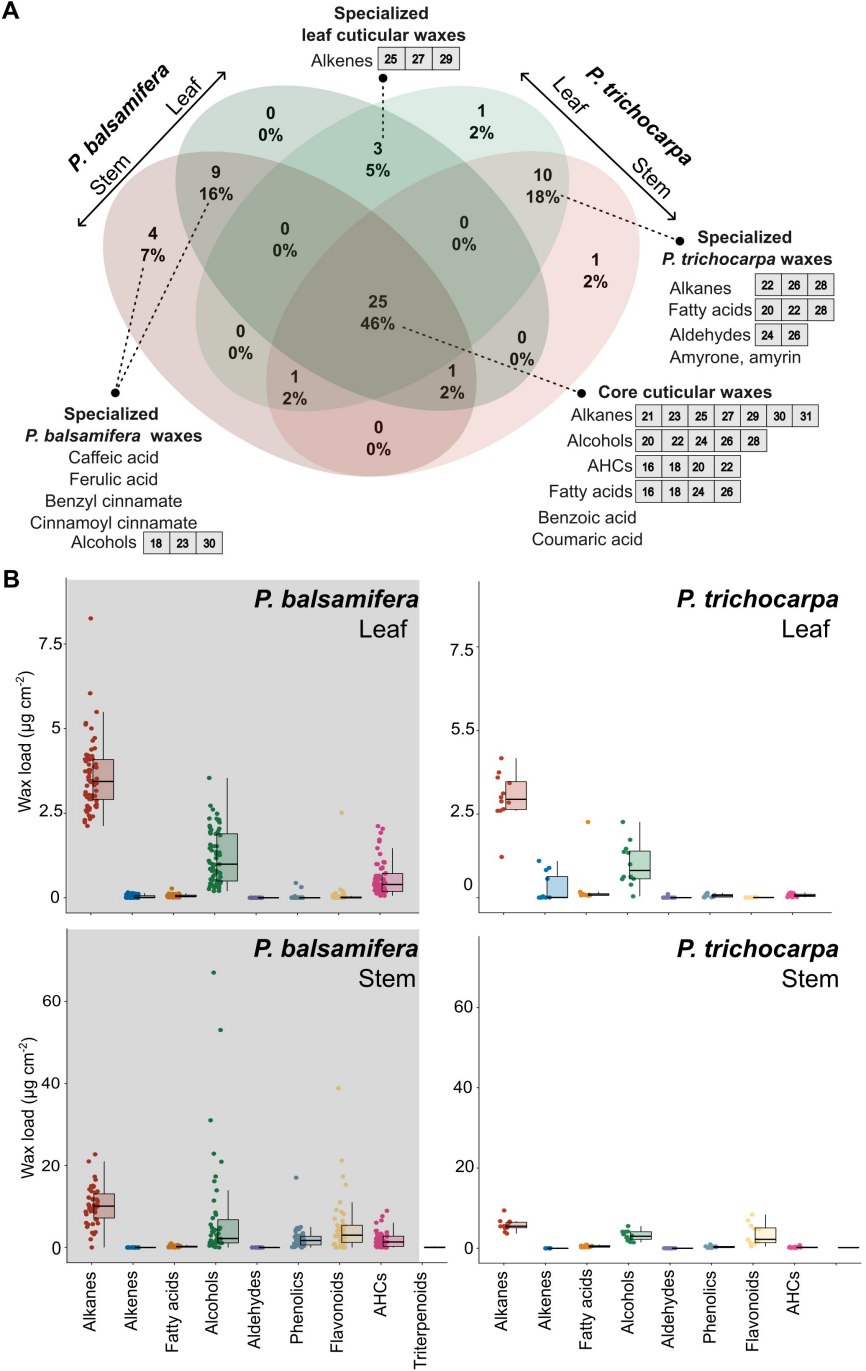
Comparison of cuticular waxes from stem and leaves of *P. balsamifera* and *P. trichocarpa.* **(A)** Venn diagram summarizing the shared and unique compounds identified in the four sample types, with specialized and core waxes listed on the sides. Numbers to the right of the compound class refer to the carbon chain length of the compounds. **(B)** Box plots representing the wax load in young stems and leaves divided into compound classes. Dots represent individual measurements of 16 *P. balsamifera* and 4 *P. trichocarpa* accessions, each with 2–5 replicates (Allen et al., 2021). Due to unequal number of accessions between species, comparisons should be interpreted with caution.

Although *P. trichocarpa* and *P. balsamifera* are closely related, they inhabit areas with different environmental conditions. To assess the degree of chemical variation, accessions from 16 P*. balsamifera* accessions and four previously characterized *P. trichocarpa* (Gonzales-Vigil et al., 2017), were grown in a growth chamber for three months and the wax profiles of leaves and stems compared. Large variation is observed in wax composition and abundance between species, and within the species depending on the type of tissue (leaf or stem), even when plants were grown under the same conditions (**Figure 1**).

A core set of cuticular wax components that are present in all tissues, accessions and developmental stages was identified (**Figure 1A**; **Supplementary Table 1**). These 25 compounds include alkanes, fatty acids, primary alcohols and AHCs. However, there are other compounds that are species- or tissue-specific, herein referred as specialized cuticular wax components. We hypothesize that specialized waxes are more likely to function in environmental adaptation and serve roles that are species- or tissue-specific. Alkenes fit within this class, being abundant in the leaves of some individuals, but undetectable in stems. Similarly, certain phenolics (caffeic and ferulic acid, cinnamoyl cinnamate, and benzyl cinnamate) were not detected in *P. trichocarpa.*

Based on their biosynthetic origin, wax components can be classified into two general classes: aliphatics derived from VLCFAs (including alkanes, alkenes, aldehydes, primary alcohols and fatty acids) and phenolic compounds derived from the core phenylpropanoid pathway (such as simple phenolics, flavonoids and AHCs). Overall, *P. balsamifera* displayed a larger phenolic-derived content in stems compared to *P. trichocarpa* (**Figure 1B**; **Supplementary Tables 2, 3**). These results highlight key differences between *P. balsamifera* and *P. trichocarpa* stem and leaf waxes.

### 3.2 Stem development influences wax accumulation

**Figure 2:**
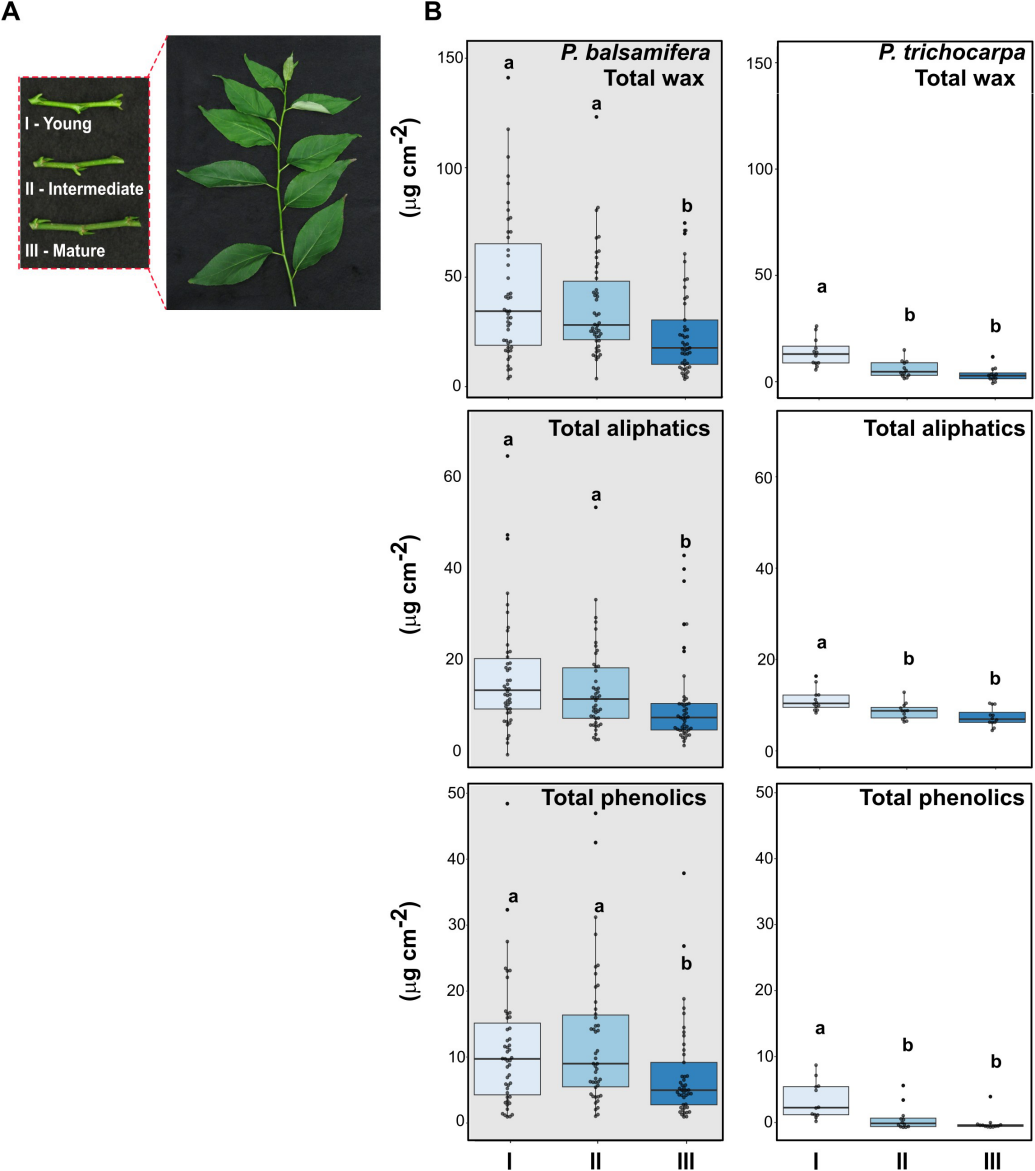
Stem cuticular wax composition at three different developmental stages in *P. balsamifera* and *P. trichocarpa.* **(A)** Representative image of *P. balsamifera* (MTN 8) showing the three developmental stages used for this analysis. I: young, II: intermediate, III: mature. **(B)** Total wax load including unknown compounds (first row), and the main two wax chemical classes of aliphatics (second row) and phenolics (third row) across 16 accessions of *P. balsamifera* (left column) and 4 *P. trichocarpa* (right column), each with 2–3 replicates. Results from Tukey (HSD) test are indicated as letters.

Stems had higher total wax compared to leaves, but it remained to be investigated if development affected the deposition of stem waxes, like it does in leaves (Chen et al., 2023; Gonzales-Vigil et al., 2017). To test this, three stem sections from three-month old plants were investigated (**Figure 2A**; **Supplementary Table 4**). The young stems of both species accumulated on average 30% more wax per unit area (**Figure 2B**). The reduction in wax load in more mature stems coincided with a reduction in phenolics, whereas aliphatics remained more uniform in both species (**Figure 2B**; **Supplementary Table 4**).

Aliphatic components commonly dominate the cuticular wax profile in plants, while aromatics (flavonoids, AHCs and other phenolic compounds) are more abundant in suberin and associated waxes (Rains et al., 2018). *P. balsamifera* stems contained on average 4 times more phenolics than *P. trichocarpa*, including larger quantities of caffeic, ferulic, *p*-coumaric, and benzoic acids, AHCs, as well as benzyl cinnamate. Moreover, variation in phenolic accumulation was observed among accessions and across developmental stages.

### 3.3 Changes in wax composition are accompanied by differences in function

**Figure 3:**
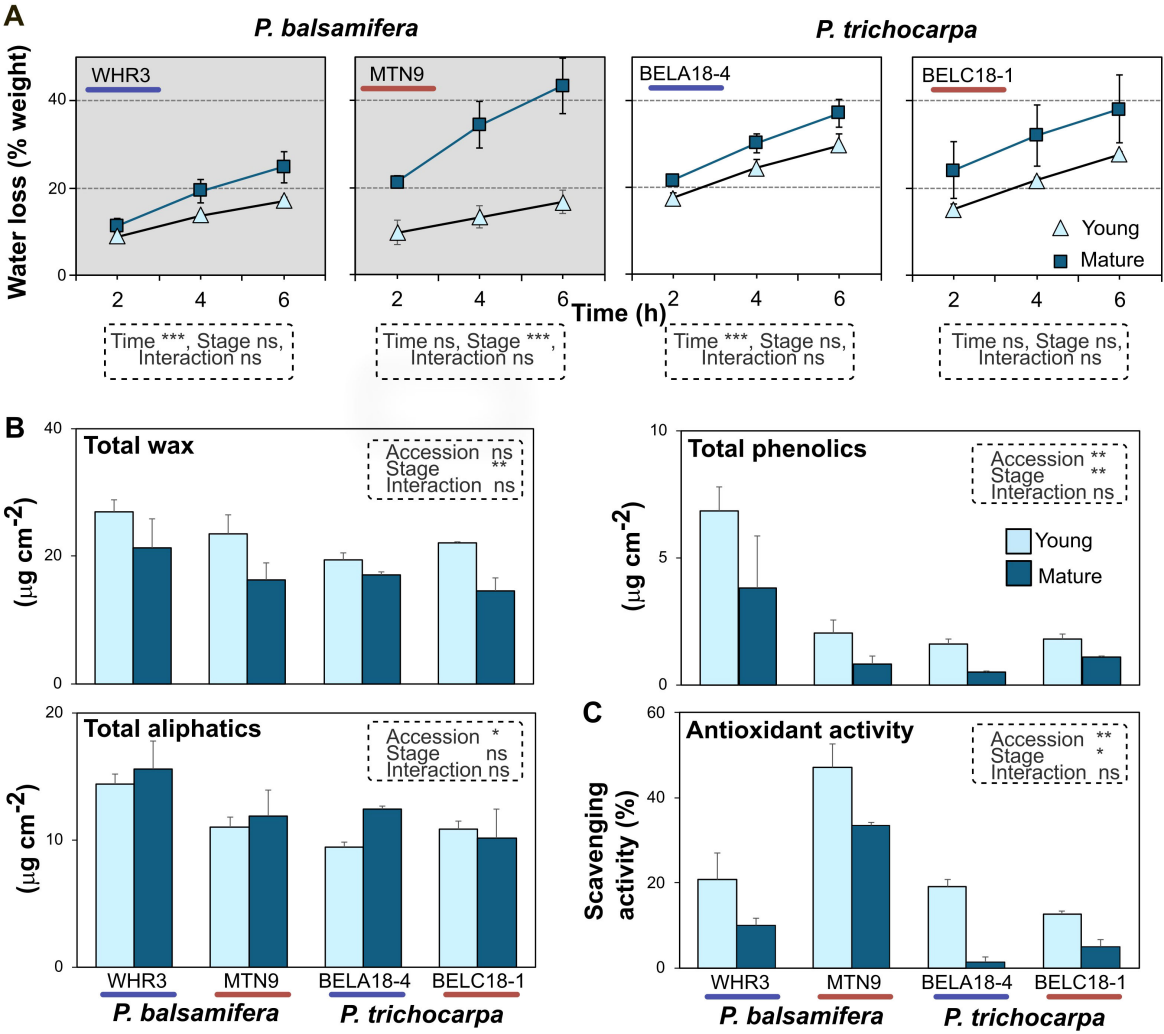
Leaf cuticular wax composition and function. **(A)** Non-stomatal water loss was measured over the course of 6 h at room temperature for young (light blue triangles) and mature (blue squares) leaves. At each time point, 3 leaves were measured. Homogeneity of variance across groups using a Levene’s test for three factors (stage, time and accession) identified significant differences in stage and accession. Results from the Scheirer-Ray-Hare test for each accession as a function of time and stage shown underneath the graphs. **(B)** Comparison of total wax content (first panel), total aliphatic (second), and total phenolics (third) waxes of the young (light blue) and mature leaves (blue). **(C)** Antioxidant activity of the wax extract from young (light blue) and mature (blue) leaves measured using reduction of DPPH method. Two accessions of each of *P. balsamifera* (WHR3 and MTN9) and *P. trichocarpa* (BELA18–4 and BELC18-1) were examined. AM accessions are underlined in blue and AP in red. Error bars represent standard error of n=3 replicates. Results from the Scheirer-Ray-Hare test indicated in the inset. *p*-value< 0.05 (*),< 0.01 (**) and< 0.001 (***).

Cuticular waxes change in quantity and composition with development, but it was unclear whether these changes affected cuticular function. Hence, we compared leaves at two developmental stages for two properties conferred by the cuticle: non-stomatal water loss measured as cuticle permeability, which has been previously associated with aliphatics, and antioxidant activity, which is attributed to phenolic components (Reynoud et al., 2021; Stanciu et al., 2023). To assess cuticle permeability, the weight of excised leaves was monitored for 2 AM and 2 AP accessions from each species over 6 hours (**Figure 3A**). Across the accessions tested, mature leaves lost more water, suggesting higher permeability compared to their younger counterparts, particularly in MTN9 (**Figure 3A**). Surprisingly, the presence of alkenes in mature leaves did not result in reduced water loss in the two AP accessions. Instead, the results suggest a negative association between total wax load with non-stomatal water loss for these four accessions (**Figure 3B**).

Cuticular waxes are not only a physical barrier against water loss, but they also provide protection from damaging reactive oxygen species (Renault et al., 2017). In this study, we identified both non-esterified phenolic compounds, like benzoic and coumaric acids, dihydroxy benzophenone and dihydroxy-methoxy-dihydrochalcone, together with esterified phenolic derivatives, such as phenyl benzoate and alkyl hydroxy coumarates (AHCs) as part of the cuticular waxes. To test if phenolics deposited on the cuticle provide antioxidant protection, we used the DPPH assay to compare leaves from two developmental stages. For all accessions, higher radical scavenging activity was observed in waxes extracted from young leaves, which have a higher phenolic content. However, the elevated antioxidant activity of MTN9 waxes was not accompanied by a higher phenolic content (**Figures 3B, C**). Conversely, WHR3 exhibited the highest total phenolic content, but it did not translate into higher scavenging activity. These observations suggest that other wax components, rather than the total phenolic content, can contribute to the antioxidant activity.

In summary, the denser and phenolic-rich wax of young leaves is more impermeable and better protected from oxidative stress. These results indicate that changes in cuticle composition across development can affect its function, preventing water loss and scavenging reactive oxygen species.

### 3.4 Genome wide analysis identifies a narrow region associated with alkene production

**Figure 4:**
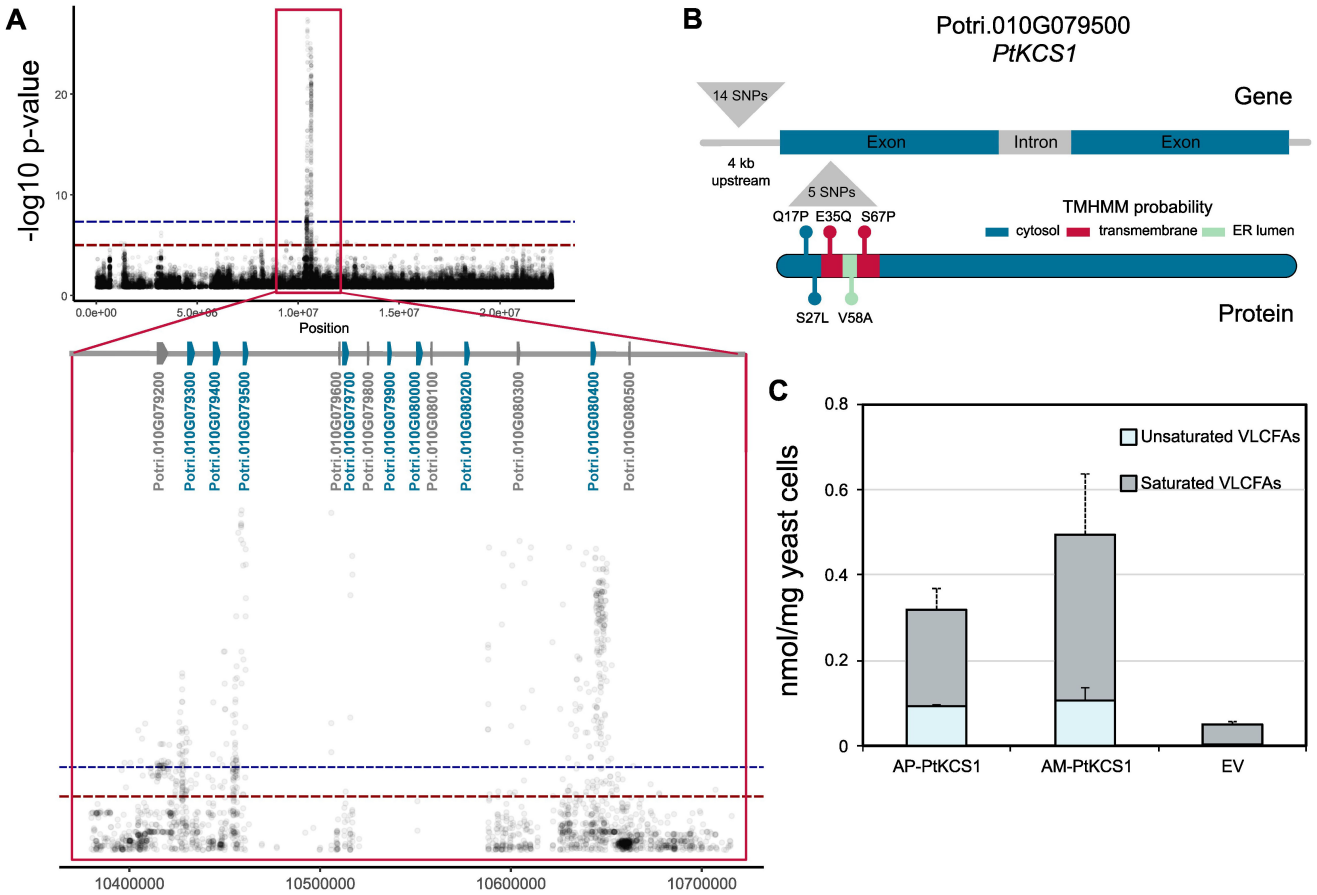
Genome-wide association analysis of alkene presence in *P. trichocarpa.* **(A)** Manhattan plot showing 332 highly-significant SNPs (out of 337) found clustered in chromosome 10. Zoom-in shows the significant SNPs lie in a region containing eight KCS-encoding genes (based on Phytozome v3.1 of the genome, shown in teal). Significance levels are indicated with lines, with the red line marking genome-wide significant SNPs at a *p*-value of 5x10^-8^, and the blue line marking suggestive associations 1x10^-5^. **(B)** Position of significant SNPs in the coding and 4 kb region upstream of *PtKCS1*, and the corresponding amino acid changes relative to their predicted subcellular localization based on TMHMM probability. **(C)** Very-long-chain fatty acid profile of yeast expressing the original PtKCS1 from an AP accession and modified to contain SNPs from AM accessions. Empty vector (EV) control provided as a reference for basal levels of VLCFA accumulation. The levels of saturated and monounsaturated fatty acids between AP- and AM-KCS1 were not significantly different (Unpaired two-tailed *t*-test analysis revealed no statistical differences in VLCFA levels between AM- and AP-PtKCS1; saturated fatty acids: t-value 1.0849, *p*-value 0.31; unsaturated: t-value 0.4203, *p*-value 0.68).

Given the strong association between *PtKCS1* expression and the accumulation of alkenes in *P. trichocarpa* (Gonzales-Vigil et al., 2017), we proceeded to search for SNPs associated with the accumulation of alkenes that could explain the low expression of *PtKCS1* in AM accessions. To accomplish this, alkene accumulation was treated as a binomial trait to conduct GWAS on 174 accessions of *P. trichocarpa*. After a conservative Bonferroni correction for multiple tests, 337 SNPs were found associated at an alpha value of 0.001. Notably, 332 of those SNPs are in a 226,623 bp region of chromosome 10 between positions 10,397,490 and 10,664,113 bp, a region that contains a cluster of eight closely-related KCS genes including the previously described *PtKCS1* in the Nisqually-1 reference genome (**Figure 4A**).

Zooming in on this region showed an uneven distribution of SNPs, with three enriched areas: one upstream of Potri.010G079300 (*PtKCS4*), another upstream of Potri.010G079500 (*PtKCS1*), and a third downstream of Potri.010G080400 (*PtKCS9*) (**Figure 4A**). To investigate if the other genes within the region are differentially expressed in AM and AP accessions, their expression was plotted as a function of the alkene phenotype (**Supplementary Figure S1**). Eight genes had low levels of expression (lower than 10 FPKM) in leaves, reducing the chances of being implicated in wax production. Of the six genes expressed, two are differentially expressed in AM and AP accessions (*PtKCS1* and *PtKCS3*), increasing the probability of being associated with alkene deposition. These close paralogs differ in only 9 bases (99% identical), confounding the mapping of the short-reads used in this study. Around *PtKCS1*, 14 significant SNPs were located 4 kb upstream of the start codon and five in the first exon leading to non-synonymous changes in the protein sequence (**Figure 4B**). The associated SNPs within *PtKCS1* are located in the first 70 residues of the protein, a region containing two transmembrane domains and far away from the catalytic site, making them unlikely to disrupt activity. To test their effect on enzyme activity, PtKCS1 from an AP accession (AP-PtKCS1) was modified to include the 5 SNPs observed in AM accessions (AM-PtKCS1). No significant differences were observed in the elongation of saturated or monounsaturated VLCFAs (**Figure 4C**). Therefore, the observed differences in *PtKCS1* expression between AM and AP accessions are more likely attributable to changes somewhere else in this region; nevertheless, these associated SNPs are strongly linked to the alkene phenotype.

**Figure 5:**
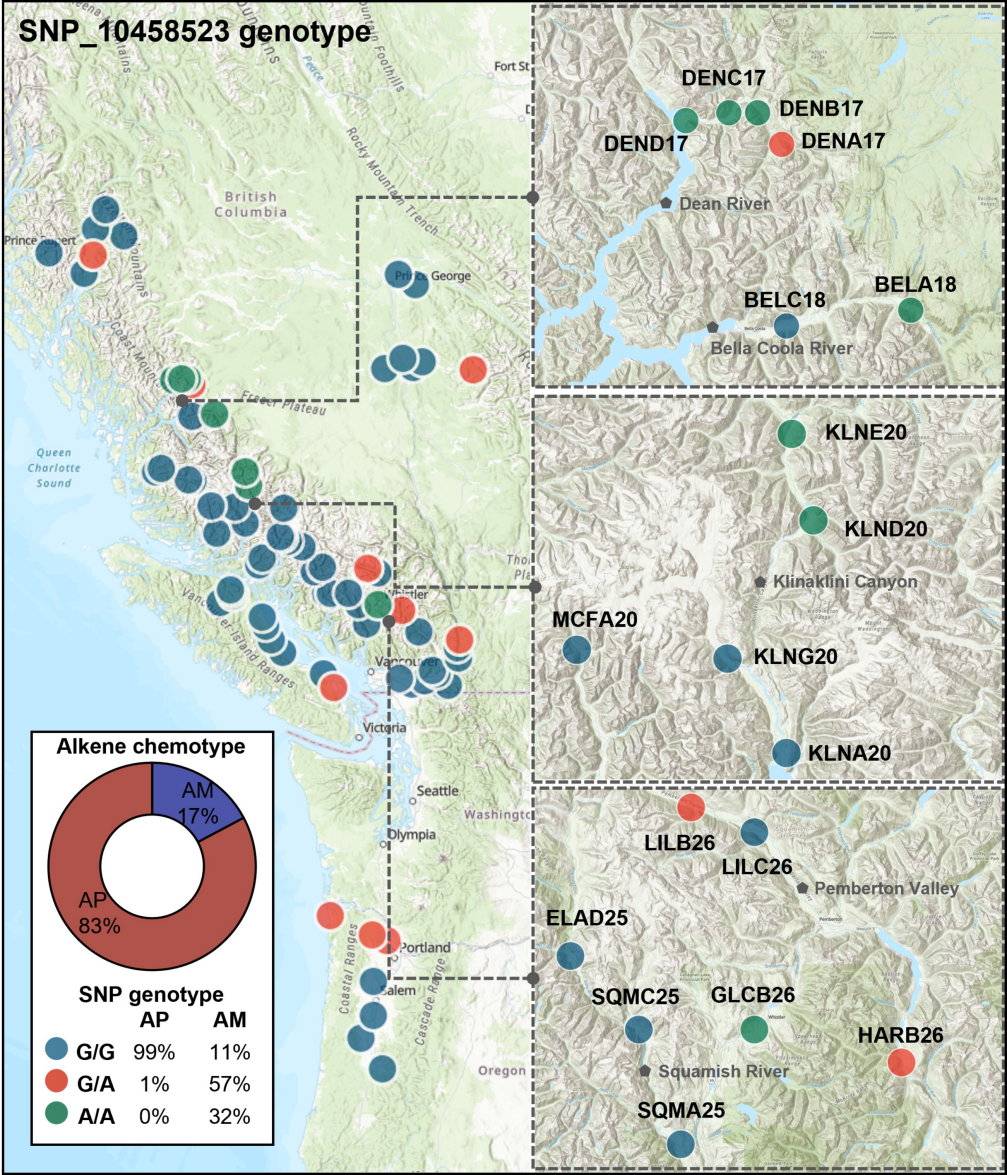
Geographic distribution of SNP predictive of alkene accumulation in *P. trichocarpa.* The SNP located at 10458523 bp on chromosome 10 is highly correlated with alkene presence with the majority of alkene-plus (AP) accessions carrying the reference allele (G/G in teal blue), whereas alkene-minus (AM) carry at least one copy of the alternate allele (G/A in red or A/A in green). Insets show the localization of the seven homozygous individuals for the alternate allele. Map created with ArcGIS.

Close inspection of the associated SNPs showed that the genotype frequency differed in AP and AM accessions (**Figure 5** and **Supplementary Table 5**). In *P. trichocarpa*, AP accessions are mostly homozygous for the reference allele (>90%), whereas AM accessions are predominantly heterozygous or homozygous for the alternate allele, suggesting that the absence of alkenes allele behaves as a dominant negative trait. Notably, only seven accessions were homozygous for the alternate allele for the SNP located at 10,458,523 bp and they do not originate from the same site (**Figure 5**).

### 3.5 Frequency of AM phenotype varies between species

**Figure 6:**
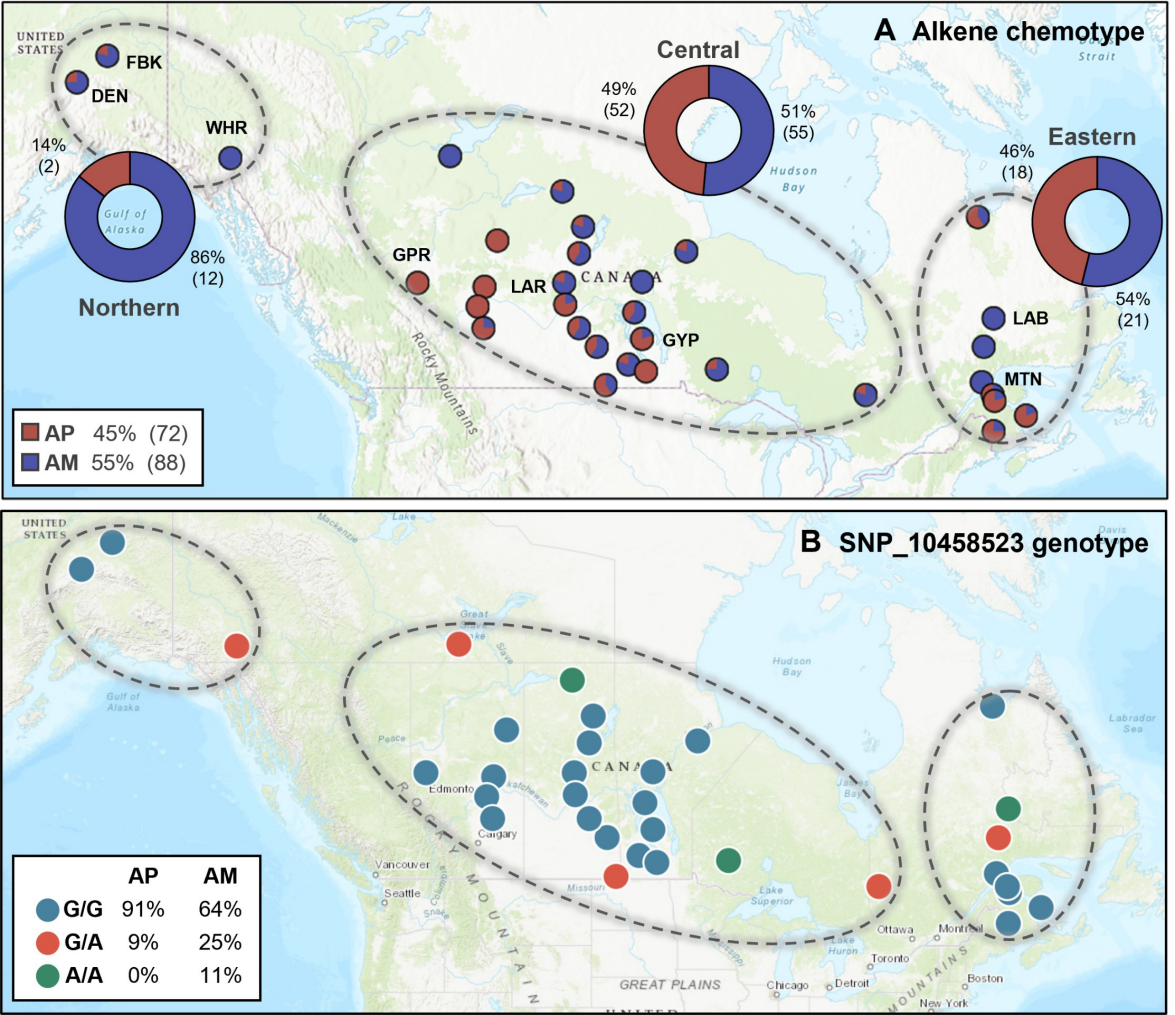
Geographic distribution of alkene-minus (AM) and alkene-plus (AP) phenotypes and genotypes in *P. balsamifera.* **(A)** Accessions from 33 provenances (160 accessions) were screened for the presence of alkenes in their leaf cuticular wax. Accessions were classified as AM (blue) if their total alkene load was below 0.02 µg/mm2 or AP (dark red) if it was higher. The total number of accessions per wax phenotype per deme is shown in the insert. **(B)** Base calling at the SNP located at position 10,458,523 bp on chromosome 10, with the reference allele being more prevalent in AP and AM accessions. Maps created with ArcGIS.

*P. balsamifera* accessions also accumulate alkenes on their leaves, albeit to lower levels than *P. trichocarpa* (**Supplementary Figure 2**). Since they are closely related species, we hypothesized that alkene accumulation might be controlled by the same genes. To investigate this, 160 accessions of *P. balsamifera* corresponding to 33 provenances were screened for their alkene content (**Supplementary Table 6**). Similar to *P. trichocarpa*, the dominant alkenes were pentacosene and heptacosene (highest load of 0.095 and 0.144 µg/mm^2^, respectively), with minor quantities of nonacosene (maximum of 0.018 µg/mm^2^). The proportion of AP and AM phenotypes differed between species, with AM comprising 17% of *P. balsamifera* accessions and 55% of *P. trichocarpa* (**Figure 5**, **6**). Notably, both chemical phenotypes were represented in each of the three previously characterized demes of *P. balsamifera* found in the Northern, Central, and Eastern range of the distribution (Keller et al., 2010).

Next, the geographical coordinates of the sites of collection were used to obtain climate data with ClimateNA revealing some differences in temperature and precipitation (Wang et al., 2016). First, AP accessions of *P. balsamifera* were preferentially distributed in areas with warmer temperatures, including higher mean annual temperature, higher warmest and coldest month temperatures (**Supplementary Figure 3**, **Supplementary Table 7**). Second, AP accessions of *P. trichocarpa* were distributed in areas that receive more precipitation, on an annual basis and during the growing period (May to September), whereas these factors were not significant in *P. balsamifera*. The Hargreaves climatic moisture deficit (CMD) can be used to assess drought risk, with higher values indicating a larger moisture deficit. In this aspect, AP accessions of *P. balsamifera* and AM accessions of *P. trichocarpa* seem to be under higher risk (**Supplementary Figure 3**, **Supplementary Table 7**). Altogether, the bioclimatic variable analysis suggests that alkene accumulation could confer an adaptive advantage, although the selection forces might be different for each species.

### 3.6 SNPs identified in *P. trichocarpa* are conserved in *P. balsamifera*, but not associated with alkene accumulation

GWAS was conducted with 138 individuals of *P. balsamifera* following the same procedure as for *P. trichocarpa.* However, no significant SNPs were found in the chromosome 10 region or elsewhere in the genome (**Supplementary Figure 4**). Surprisingly, the highly-significant SNPs found in *P. trichocarpa* are also present in *P. balsamifera*, but their allele frequencies differ between species (**Supplementary Figure 4**, **Supplementary Table 5**). In *P. balsamifera*, most AP and the majority of AM accessions are homozygous for the reference allele (91% and 64%, respectively), whereas the alternate allele is more frequent in AM than AP accessions (**Figure 6**). Therefore, even though these positions are excellent markers linked with alkene accumulation in *P. trichocarpa*, their predictive power is reduced in *P. balsamifera*.

## 4 Discussion

### 4.1 Connecting function with cuticular wax variation

In this study, we identified qualitative and quantitative variation in waxes across tissues and throughout development. To disentangle the chemical variation, cuticular wax components were classified based on their presence/absence in stem and leaves of *P. trichocarpa* and *P. balsamifera* leading to separation into core and specialized cuticular waxes. Core cuticular waxes, found in both tissues and species, include mostly aliphatic components (alkanes, alcohols, fatty acids) and AHCs; and are likely responsible for the primary functions of the wax in water retention. In contrast, alkenes are specialized leaf waxes not detected in stems. Additionally, development played an important role in wax deposition with stems from 3-month-old plants consistently accumulating a higher wax load per surface area in younger sections. This matches previous observations in leaves of *P. trichocarpa* (Gonzales-Vigil et al., 2017), and could indicate higher metabolic activity in wax biosynthesis in younger tissues or that the rate of area expansion is higher than the rate of wax deposition in more mature stages. The key role of developmental stage on cuticular waxes has been reported in other *Populus* species (Cameron et al., 2002; Gonzales-Vigil et al., 2017; Grünhofer et al., 2021a; Rains et al., 2018), and could be explored to uncover master regulators of wax deposition.

Large differences were also observed in phenolic compounds, which varied between and within species, and with developmental stage. Specifically, phenolics show higher accumulation in cuticular waxes of younger tissues compared to their mature counterparts, unlike the fairly constant accumulation of most aliphatics (Chen et al., 2023). These observations could suggest specialization of the waxes given the functional differences between aliphatics and phenolics, or a trade-off between these classes driven by competition for carbon allocation in epidermal cells. Phenolic compounds are credited with protection against oxidative stress (Burchard et al., 2000; Shahidi and Chandrasekara, 2009), hence it is possible that tissues more exposed to light, as younger tissues usually are, require heavier deposition to reduce damage from oxidation. In line with this hypothesis, the antioxidant activity of wax extracts was higher in younger leaves in both poplar species. However, phenolic content and antioxidant activity were not perfectly correlated. WHR3 exhibited the highest total phenolic from the four accessions; yet, it was MTN9 which had the highest scavenging activity. Interestingly, the larger phenolic content of WHR3 was mostly due to a high accumulation of AHCs (**Supplementary Table 2**), which may indicate that individual compounds may have differential scavenging attributes. This is in line with previous studies indicating that even non-phenolic molecules can confer antioxidant properties (Foti and Amorati, 2009).

While previous studies have reported that the phenolic compounds extracted from poplar buds have quenching activities (Dudonné et al., 2011; Stanciauskaite et al., 2021), research on the antioxidant properties of the wax extract specifically in poplar species is still scarce. Protecting internal tissues from desiccation is the most well-known function of the cuticle. However, whether this property is conferred by the amount of total wax or its composition is still under scrutiny. In *P. euphratica*, higher wax load results in decreased cuticle permeability (Xu et al., 2016). Other studies have pointed at alkanes as the components strongly influencing permeability and non-stomatal water transpiration (Negin et al., 2023). However, these observations seem to be context dependent. For example, a ten-fold increase in total wax did not affect residual transpiration in *P.× canescens* (Grünhofer et al., 2022b). A follow-up study using a mutant with altered wax composition and normal wax load, demonstrated that composition better predicted water loss through the cuticle (Grünhofer et al., 2023). Furthermore, a recent study comparing the cuticular waxes of 339 natural accessions of *P. trichocarpa* grown under control and drought conditions in a common garden, found no major differences in total wax load upon drought conditions; however, the contribution of certain wax components to the total was indeed responsive to drought (Karaca-Bulut et al., 2025). We initially hypothesized that older leaves would have a more impermeable cuticle given the larger contribution of aliphatics to the wax, and that alkene deposition might provide additional protection to AP accessions. In contrast, wax quantity, regardless of whether the components are phenolics or aliphatics, was a better predictor of permeability in the four accessions tested. Specifically, mature leaves lost more water compared to younger ones, irrespective of the species or production of alkenes. This matches previous reports that AP accessions of *P. trichocarpa* have lower water-use efficiency compared to the AM accessions (Gonzales-Vigil et al., 2017), suggesting that alkenes do not contribute to the impermeability of the cuticle.

In summary, developmental changes in wax composition within the same plant can impact the functions of the cuticle. It is possible that the chemical differences observed across the geographic distribution of the species might also be affecting function. Supporting this, the alkene accumulation phenotype showed different of geographic distribution in *P. trichocarpa* and *P. balsamifera*, with differences noted as a function of precipitation and temperature. Since cuticular waxes are associated with an array of functions, here we propose that specialized cuticular waxes that are exclusive to a species or tissue are more likely to provide an adaptive advantage, and hence should be prioritized to test for function.

### 4.2 Variation in the distribution of the alkene-producing phenotype

Alkene-producing phenotypes are predominant in *P. trichocarpa*, but not in *P. balsamifera*. We have previously reported that *P. trichocarpa* AM trees tend to grow smaller in the common garden with mild and humid conditions (Gonzales-Vigil et al., 2017). This coincides with the fact that AM accessions originate from areas that receive less precipitation than AP collection sites. It is tempting to speculate that AM is a maladaptive trait in the range of distribution of *P. trichocarpa*, leading to the dominance of the AP phenotype. In contrast, there are populations fixed for AP and AM in *P. balsamifera*, suggesting that the trait is neutral in its range of distribution. However, significant associations were detected between the climatic variables of sites of collection and the alkene phenotype in *P. balsamifera*, most notably, mean annual temperature.

Adding to the complexity is the fact that these two species can hybridize, with gene flow biased towards introgression of *P. balsamifera* genes into *P. trichocarpa* (Dickmann et al., 2001; Thompson et al., 2010; Suarez-Gonzalez et al., 2016). Hybridization occurs extensively in Alaska, northwestern Canada, and the Canadian Rockies (Dickmann et al., 2001; Geraldes et al., 2014). Although AM and AP individuals are evenly distributed in the Central population of *P. balsamifera*, accessions closer to the sympatric zone are predominantly AP. Interestingly, GPR1 (AP) has previously been reported as an admixed *P. balsamifera* with genomic content from *P. trichocarpa* (Geraldes et al., 2013). To investigate this possibility further, we interrogated the highly significant SNPs from *P. trichocarpa* in the chromosome 10 region in *P. balsamifera*, finding the same polymorphisms.

The most parsimonious explanation is that the reference and alternate allele were present in the common ancestor of *P. balsamifera* and *P. trichocarpa*, and maintained by balancing selection. The Central deme is considered closer to the ancestral population in *P. balsamifera* (Keller et al., 2010). If both alleles were present in the Central refugia before the last glaciation event, they would have been maintained as they re-colonized Northern and Eastern environments. Alternatively, introgression of the reference allele into *P. balsamifera* or of the alternate allele into *P. trichocarpa* could have occurred at certain locations. If this were the case, a higher frequency of the alternate allele in proximity to the contact zones would be expected in *P. trichocarpa*. However, such pattern was not observed. *P. trichocarpa* individuals that were homo- or heterozygous for the alternate allele were rather localized around the Coast Mountains, away from the hybridization zones. The accessions found closer to the sympatric zones were predominantly homozygous for the reference allele, except for KIMB16-3. Similarly, *P. balsamifera* individuals can hybridize at Grand Prairie (GPR), Fairbanks (FBK), Denali National Park (DEN) and Whitehorse (WHR). Interestingly, the reference allele from *P. trichocarpa* was also found in regions as far away as Matane (MTN) in the East. This could be due to introgression of *P. trichocarpa* into Québec breeding materials in early 1970s (Vallée, 1975), or presence of both alleles in the ancestral population.

Reciprocal transplants of AM and AP phenotypes from both species would be required to determine if alkenes provide a selective advantage under contrasting environmental conditions, particularly in terms of temperature and precipitation.

### 4.3 Genetic basis of alkene accumulation

The availability of SNPs in these species enabled investigating the genetic basis of alkene accumulation. Treating alkene accumulation as a discrete trait with two possible phenotypes led to the identification of 332 SNPs associated with the trait in *P. trichocarpa*. Despite this being a genome-wide scan, almost all significant SNPs were clustered around a narrow region on chromosome 10. The analysis clearly pointed to the loci harboring the KCS cluster on chromosome 10 as responsible for the accumulation of alkenes in *P. trichocarpa* accessions. However, the SNPs found in the coding region of *PtKCS1* are not likely responsible for the low expression, and clearly do not affect enzyme activity. Notably, a recent study used long-read sequencing to assemble chromosome 10 from 39 accessions of *P. trichocarpa*, finding additional copies of KCSs in this region relative to the reference Nisqually-1 genome (Kainer et al., 2026). In that case, five out of the six AM individuals carried a nonfunctional copy of *PtKCS1*; however, the number of copies of *PtKCS7* was a better predictor of the alkene phenotype. Given the large number of highly similar KCS genes interspersed with transposons in this region, automatic assembly of reads is extremely challenging requiring additional manual curation (Kainer et al., 2026). Our approach relied on SNPs mapped with short-reads; however, indels, re-arrangements, or copy number variants that are not detected by GWAS could explain the differences in expression between AM and AP. The fact that these changes are likely in extensive linkage disequilibrium with one another adds a layer of complexity to finding the genetic cause of the AM phenotype. Yet, the mechanism responsible has to account for the observation that the alternate allele is dominant negative, where carrying one copy is enough to reduce the expression of *PtKCS1*. Interestingly, the KCS cluster on chromosome 10 was identified in a study that looked at structural variants among *P. nigra*, *P. deltoides*, and *P. trichocarpa*. Copy number variants and insertions associated with LTR Gypsy DNA transposable elements were found in this region (Pinosio et al., 2016). More recently, this same region was found strongly associated with stomatal size and abaxial contact angle in a GWAS study of *P. trichocarpa (*Klein et al., 2025). Although the SNPs identified here are great makers for the absence of alkenes, the genetic cause for the low expression remains elusive.

Failure to detect significantly associated SNPs in *P. balsamifera* with the same approach could indicate differences in the genetic control of alkene accumulation in *P. balsamifera*. Treating alkene accumulation as a binomial trait was successful in *P. trichocarpa;* however, other approaches might be required to understand the genetic regulation of alkene production in *P. balsamifera*. Several explanations are possible, one is that the genetic background is affecting the expression of the alkene phenotype differently in the two species, or that it is a more complex quantitative trait in *P. balsamifera*. The abovementioned studies have detected copy number variants around this region in *P. trichocarpa* and other more distantly related *Populus*. Hence it is plausible that similar rearrangements exist in the *P. balsamifera* pangenome. Because *P. balsamifera* has lower levels of accumulation of alkenes, a few of the homozygous individuals for the reference allele might have been mistakenly assigned to the AM class. Future studies are required to investigate alkene accumulation and expression of the tandem cluster of KCS in leaves of different developmental stages, and under contrasting environmental conditions to understand the role of *PtKCS1* and other KCSs nearby in *P. balsamifera*. The recent advances in the *Populus* pangenome with long-read sequencing techniques will be crucial in elucidating the remaining questions.

In summary, we investigated the natural diversity of cuticular wax composition among two closely related poplar species and found that environmental conditions at the site of origin likely influence this variation. Our comprehensive metabolite profiling of cuticular waxes offers valuable insights into wax biosynthesis and its functional significance, demonstrating that variation in wax composition can affect plant physiological responses, potentially contributing to adaptation to different environmental conditions. These findings provide a foundation for selecting *Populus* accessions with enhanced resiliency and suitability to future climate scenarios.

## Data Availability

Publicly available data were analysed in this study. Genomic data can be found at the NIH Sequence Read Archive as projects PRJNA276056 and PRJNA298917.
