# Supplementary material for: Cuticular wax profiling of Populus trichocarpa and P. balsamifera reveals surface similarities with underlying differences
Source: Front Plant Sci. 2026 Jun 11;17:1846385. doi: 10.3389/fpls.2026.1846385 (PMC13294658; doi:10.3389/fpls.2026.1846385)
Supplement: Supplementary file 1 [file DataSheet1.docx]

Supplementary Material

## Supplementary Figures


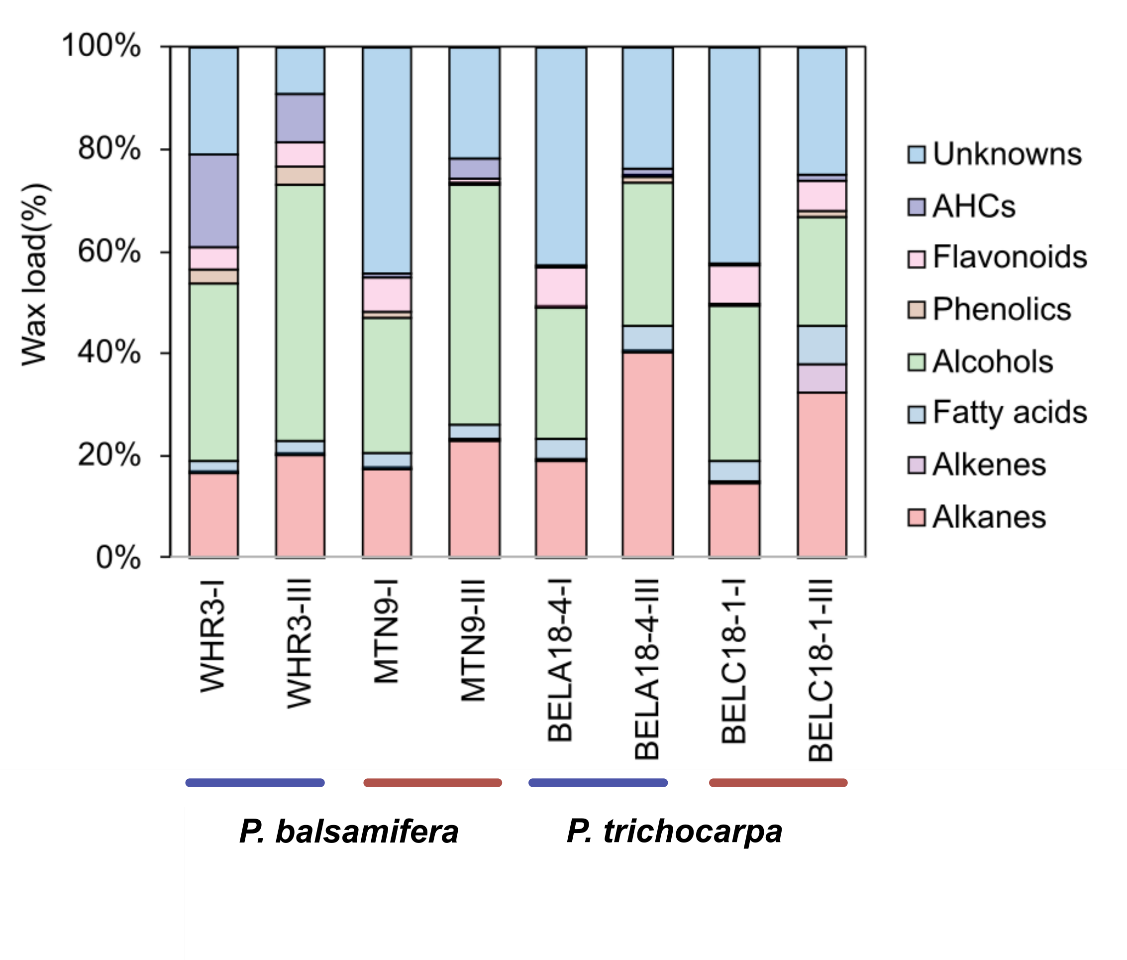


**Supplementary Figure S1.** **Percent composition of cuticular waxes in leaves.** Content of samples used for Figure 3 plotted by chemical classes and percent contribution to the total wax load. Two accessions of each of *P. balsamifera* (WHR3 and MTN9) and *P. trichocarpa* (BELA18-4 and BELC18-1) were examined. AM accessions are underlined in blue and AP in red.


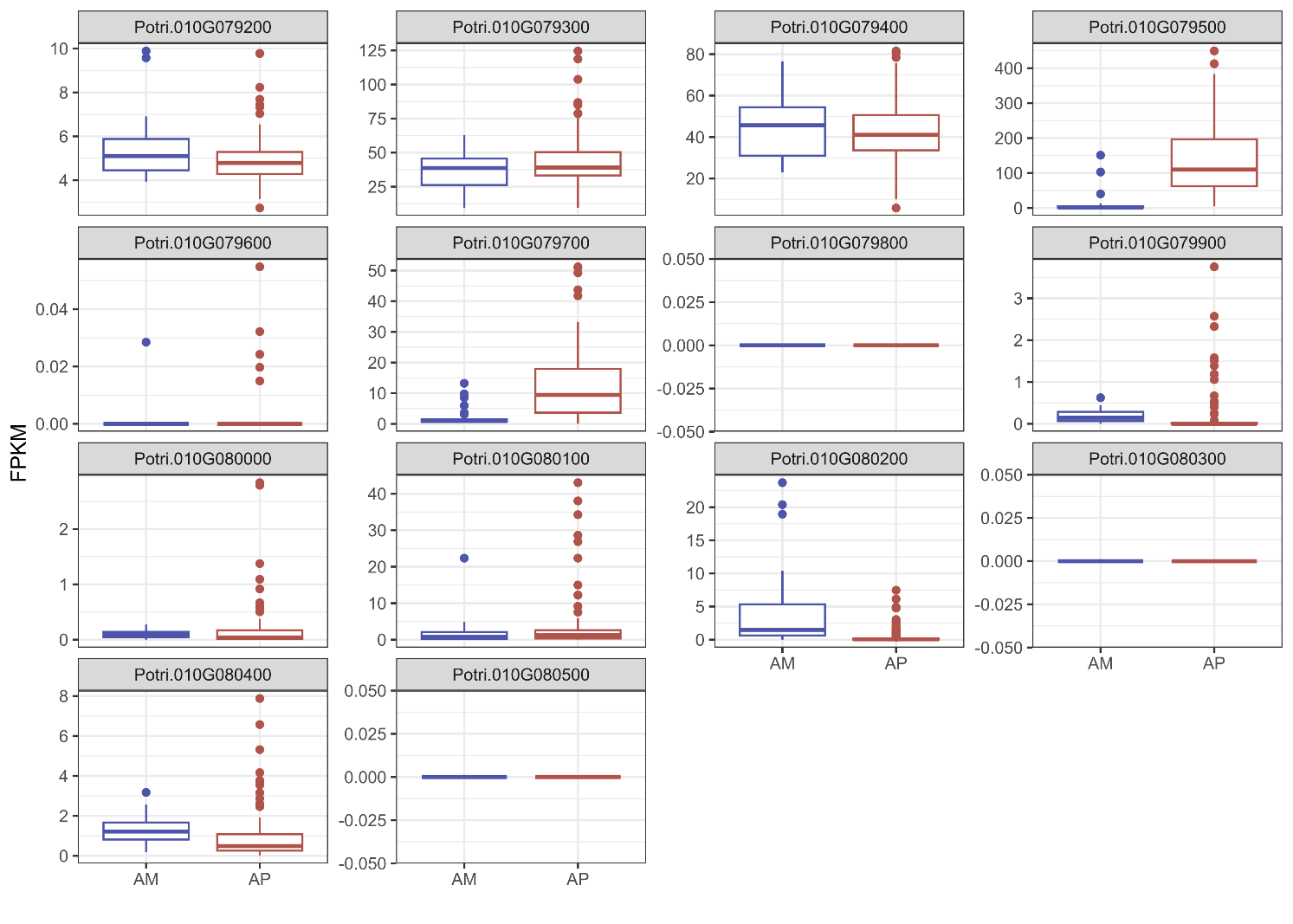


**Supplementary Figure S2.** **Transcript levels of genes within the associated region identified in the genome-wide association analysis in *P. trichocarpa* leaves.** Boxplots showing leaf expression of the predicted genes within the region identified by 332 significant SNPs (see Fig.4) in fragments per kilobase per million fragments mapped (FPKM). The 136 accessions are grouped according to their alkene phenotype (AM: Alkene minus in blue, and AP: Alkene plus in red). Outliers are shown as individual dots.

**
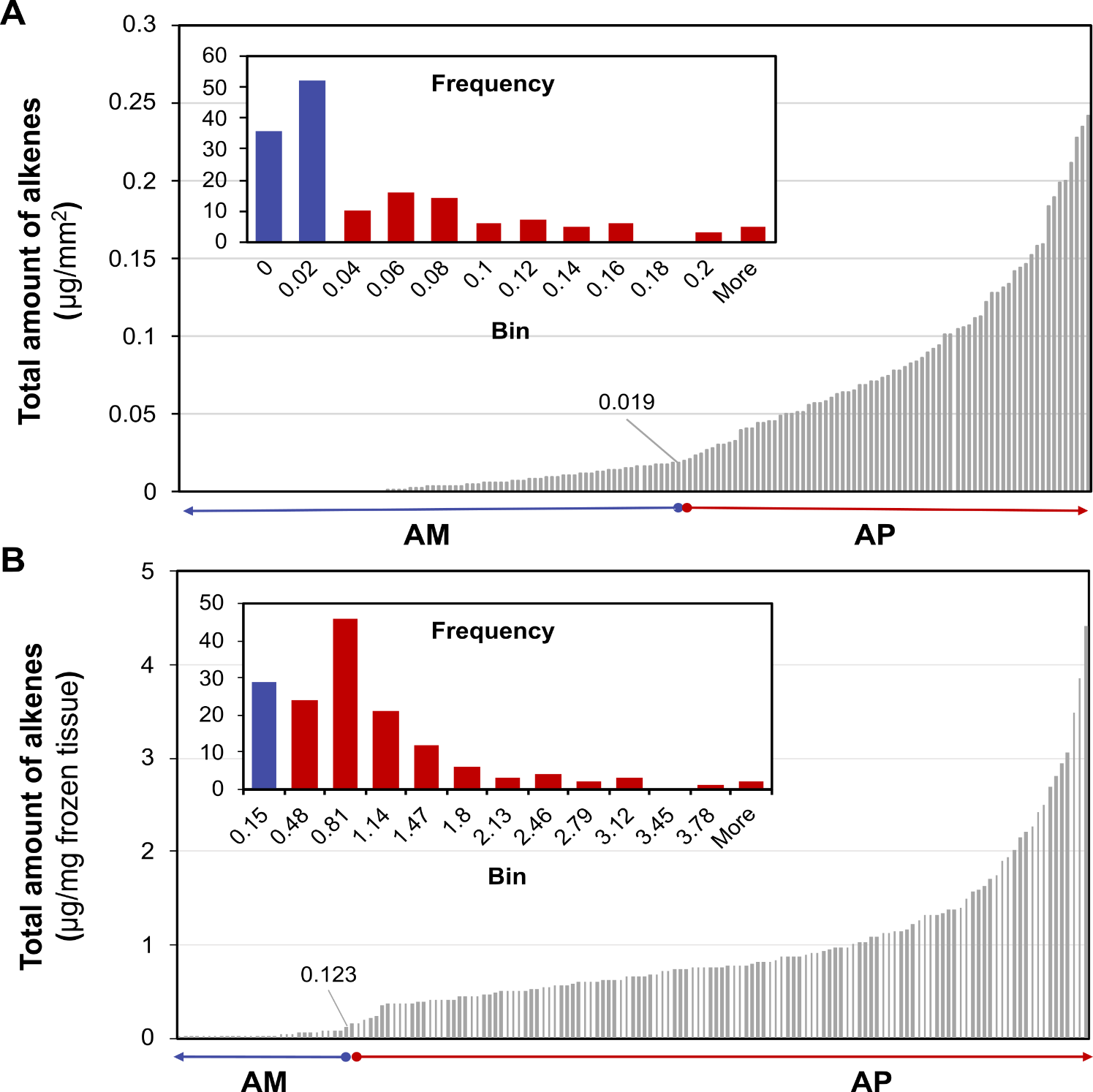
**

**Supplementary Figure S3.** **Distribution of total amount of alkenes in accessions used in GWAS.** Cuticular waxes from 160 *P. balsamifera* (A) and 154 *P. trichocarpa* (B) accessions were extracted and quantified on a GC-FID system. Leaf discs were used for *P. balsamifera* and the peak areas were normalized to the internal standard and leaf area. For *P. trichocarpa*, frozen tissue was utilized and hence the normalization is performed by the internal standard and weight of the frozen tissue. Insets show the histogram of the distribution, with the number of accessions indicated in the y-axis (AM: Alkene minus in blue, and AP: Alkene plus in red).


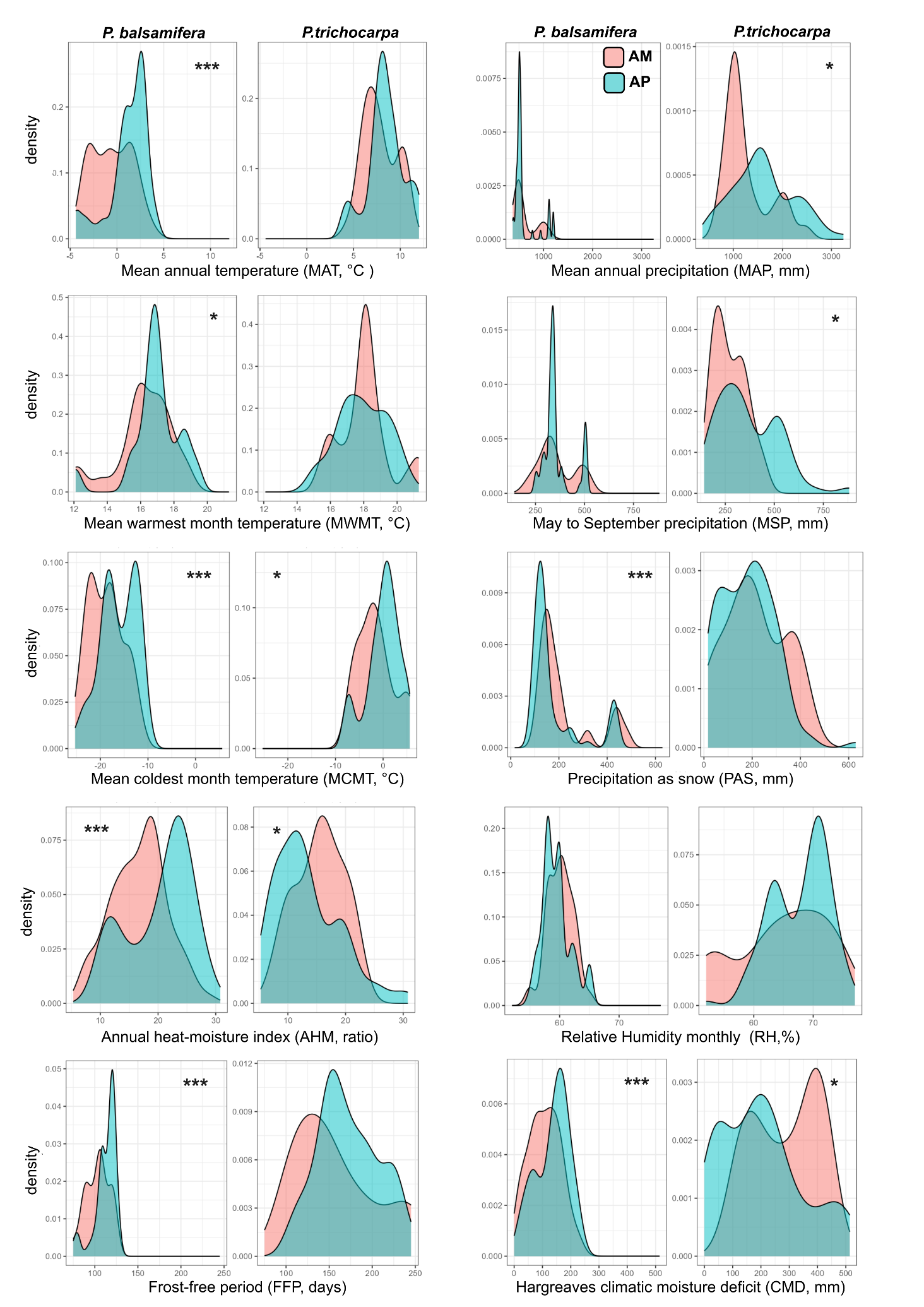


**Supplementary Figure S4.** **Bioclimatic variables at the collection sites of *P. balsamifera* (left) and *P. trichocarpa* accessions (right).** Information on bioclimatic variables was obtained from ClimateNA v5.30 (Wang et al., 2016) for the original site of collection of the accessions. Results of the adjusted p-value using the FDR for a Krustal-Wallis test comparing the averages between alkene phenotypes (AM: Alkene Minus in pink, and AP: Alkene Plus in teal) (p-adj < 0.05 (*), p-adj < 0.001 (***).


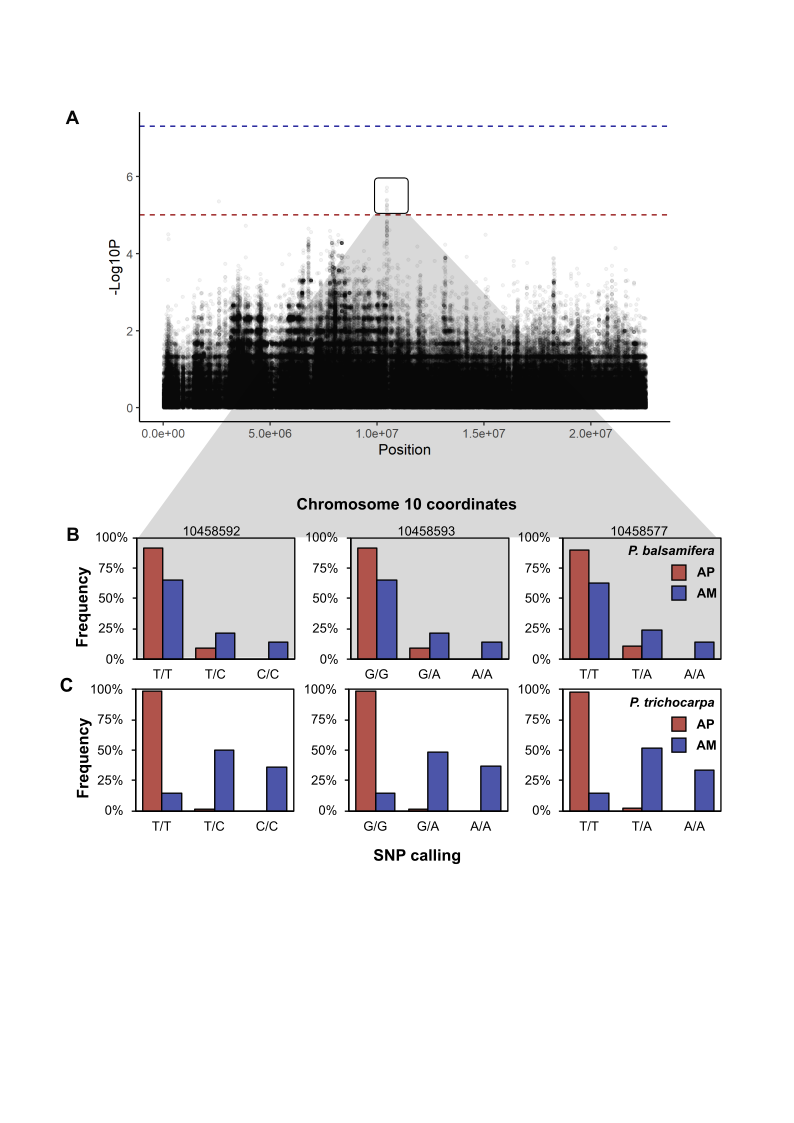


**Supplementary Figure S5.** **Genome-wide association analysis of alkene presence in *P. balsamifera*.** (**A)** Manhattan plot of chromosome 10 showing the distribution of SNPs. Eleven SNPs around the area identified in *P. trichocarpa* had a slightly elevated signal in *P. balsamifera*, but were not statistically significantly after multiple-testing correction. Comparison of the distribution of the reference and alternate allele for the three most significant *P. trichocarpa* SNPs associated with alkene accumulation in Alkene-Minus (AM: blue) and Alkene-Plus (AP: red) accessions of *P. balsamifera* (**B)** and *P. trichocarpa* (**C).**

**Wang T, Hamann A, Spittlehouse D, Carroll C**. **2016**. Locally Downscaled and Spatially Customizable Climate Data for Historical and Future Periods for North America. *PLOS ONE* **11**: e0156720.
